# Tip‐Induced 3D Printing on the Nanoscale with Field Emission Scanning Probes

**DOI:** 10.1002/smll.202409035

**Published:** 2024-12-18

**Authors:** Mathias Holz, Martin Hofmann, Frances I. Allen, Christoph Weigel, Steffen Strehle

**Affiliations:** ^1^ Institute of Micro‐ and Nanotechnologies MacroNano Microsystems Technology Group Technische Universität Ilmenau Max‐Planck‐Ring 12 98693 Ilmenau Germany; ^2^ Department of Materials Science and Engineering University of California Berkeley CA 94720 USA; ^3^ National Center for Electron Microscopy Molecular Foundry Lawrence Berkeley National Laboratory Berkeley CA 94720 USA

**Keywords:** 3D printing, bottom‐up, electron‐beam, nanostructure

## Abstract

3D printing down to the nanoscale remains a significant challenge. In this paper, the study explores the use of scanning probes that emit low‐energy electrons (<100 eV) coupled with the localized injection and electron‐induced decomposition of precursor molecules, for the precise localized deposition of 3D nanostructures. The experiments are performed inside the chamber of a scanning electron microscope (SEM), enabling the use of the in‐built gas injector system (GIS) with gaseous naphthalene precursor for carbon deposition, as well as immediate inspection of the deposits by SEM. Substrate materials are planar fused silica with thin conductive coatings and non‐planar copper wedges. After investigation of the deposition process parameters, various 2D and 3D carbon deposits are grown. Vertical nanowires several microns in length with a diameter <100 nm are achieved and 3D deposits with a high degree of nanoscale branching are also obtained, presumably due to a charging effect. High aspect ratio carbon nanostructures such as those demonstrated here can be employed as miniaturized electrodes or field emitters. The tip‐based approach presented thus paves the way toward 3D nanoscale printing of various materials and functional nanostructures.

## Graphical abstract

This paper investigates the use of scanning probes emitting low‐energy electrons (<100 eV) in combination with the local injection and electron‐induced decomposition of precursor molecules for the precise local deposition of 3D nanostructures. The presented tip‐based approach paves the way toward 3D nanoscale printing of various materials and functional nanostructures.

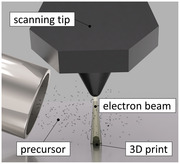

## 1 Introduction

The controlled fabrication of nanostructured surfaces and individual 3D functional nanostructures remains a significant challenge for advancing innovations in various fields, including biomedical, automotive, chemical, and semiconductor applications.^[^ ^1^ ^]^ While various 3D printing technologies have been developed and intensively studied for macroscopic and microscopic applications (e.g., fused deposition molding, selective laser sintering and two‐photon‐polymerization^[^ ^2^, ^3^, ^4^ ^]^), these cannot currently deliver the spatial resolution and geometric accuracy required for printing at the nanoscale. Here, technologies like focused ion and electron beam induced etching or deposition, which can directly create 3D structures with sizes in the micro‐ and nanoscale range, offer a promising approach for further advances in this field.^[^ ^5^ ^]^

Electron beam induced deposition (EBID) with a focused scanning electron beam was already demonstrated several decades ago. For example, using a 45 keV electron beam, surface hydrocarbon contamination was polymerized onto a thin free‐standing substrate achieving line widths below 10 nm.^[^ ^6^ ^]^ In this case, the narrow linewidth was enabled by the minimized beam backscatter due to the free‐standing sample geometry. Since then, many works have shown that by choosing an appropriate gaseous precursor, either localized deposition or gas‐assisted etching can be realized.^[^ ^7^, ^8^, ^9^ ^]^

To avoid impairment of the beam spot size due to poor vacuum, the gaseous precursors for deposition and etching are typically injected locally toward the beam illumination spot on the sample using a gas injection needle inside the SEM chamber.

During gas‐assisted etching, the decomposition products of the adsorbed precursor gas molecules react chemically with the substrate surface forming volatile compounds that result in material removal. Whereas for material deposition, the non‐volatile reaction products from the interaction of the primary beam with the precursor form highly localized deposits. By controlling the position of the beam, 3D nanostructures can thus be created. As revealed in early work, deposits can also form directly from beam‐induced interactions with contamination in the chamber atmosphere (also known as contamination lithography).^[^ ^10^, ^11^, ^12^, ^13^ ^]^ However, much greater control over the process and access to a wide range of precursor chemistries for both deposition and etching is enabled by the gas injection approach.^[^ ^9^, ^14^, ^15^ ^]^

As an alternative to using the electron beam of the SEM, the electrons required for the decomposition of the gaseous precursor molecules can also be obtained via field‐emission from nanoscale tips, as for instance shown in our previous research.^[^ ^16^, ^17^ ^]^ In comparison to traditional EBID systems, tip‐based electron beam induced deposition (TEBID) systems can be more compact and deliver much lower energy electrons (<100 eV) even under atmospheric conditions. As such, TEBID also opens the door to fundamental studies of electron‐gas interactions in the low energy regime. Studies of EBID boundary conditions in fact show that deposition processes are mainly driven by sub‐20‐eV secondary electrons, rather than by the primary keV electron beam of the SEM.^[^ ^18^, ^19^ ^]^

Therefore, the range of gaseous precursors used for EBID should also be compatible with TEBID, such as naphthalene, trimethyl(methylcyclopentadienyl)platinum(IV) (TMP) and tetraethylorthosilicate for carbon, platinum(‐carbon) and silicon‐oxide deposition, respectively. Thus, the deposition of metallic, semiconducting, and insulating materials should be possible.^[^ ^20^, ^21^ ^]^ Albeit in the case of insulating materials, additional challenges will exist, since TEBID relies on field emission and hence sufficient conductivity of the surface. For TEBID of insulating materials, 2D deposits should therefore be easier to realize than 3D deposits S1.

TEBID thus targets a similar range of applications to EBID, but the TEBID approach offers larger degrees of freedom with respect to the design and operation of the overall system, including access to higher working pressures and extension to multiple emission tips for increased throughput. When printing on sensitive materials, such as 2D materials, low‐energy operation is also expected to be beneficial. TEBID could therefore offer nanoscale 3D printing capabilities that complement the general 3D printing portfolio, enabling new device architectures with nanoscale 3D elements for future electronics including nanoelectromechanical systems, 3D electrodes, and terahertz antennas.^[^ ^22^, ^23^, ^24^, ^25^ ^]^

**Figure 1:**
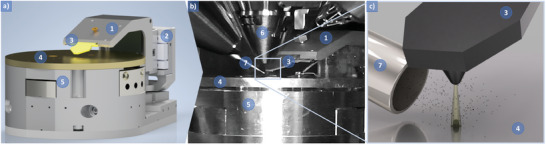
a) Schematic of the AFM that was installed in a SEM and used for the in‐situ tip‐based electron beam induced deposition (TEBID) of this study; b) SEM chamber view showing the integrated AFM system from a) – image captured with the integrated CCD camera; c) illustration of the deposition process with injected precursor molecules decomposed by the low‐energy electrons that are field‐emitted from the sharp cantilever tip. 1) Cantilever arm with read‐out electronics, 2) three axis coarse positioning unit, 3) cantilever with carrier printed circuit board, 4) sample holder supplying the bias voltage, 5) three‐axis scanning unit, 6) pole piece of the SEM column; 7) gas injector system.

With a view toward 3D nanoscale printing, we have in the present work explored the implementation of field‐emission scanning probes to create nanostructures by moving the nanoscale tips in three dimensions in a controlled trajectory during field‐emission from the tip. The TEBID system used was installed in a SEM chamber to exploit the integrated GIS and to enable immediate inspection of the tip position and the sample surface (see **Figure** **1**). Naphthalene (C_10_H_8_) was chosen as an available and well‐characterized precursor for carbon deposition^[^ ^26^, ^27^ ^]^ and was used here for the systematic creation of our 3D carbon‐based micro‐ and nanostructures. Preliminary experiments were also performed using TMP as the precursor, demonstrating the applicability of TEBID to deposit other material chemistries (see supplementary information). Using the TEBID approach, we demonstrate the reproducible fabrication of 2D and 3D deposits such as spirals, nanowire‐like and branched tree‐like nanostructures, paving the way toward tip‐based 3D material nanoscale printing from the gas phase.

## 2 Experiments, Results, and Discussion

### 2.1 TEBID System Set‐Up

For the electron emitter, the nanoscale tip of a self‐sensing and self‐actuating atomic force microscope (AFM) field‐emission cantilever was employed, as described in further detail in ref. [28, 29]. A conductive substrate is required to serve as the counter electrode in the electron field emission process. Therefore, to minimize the potential detrimental influence of semiconducting substrates (e.g., monocrystalline silicon wafers), we used fused silica substrates that were coated with a metallic layer. Specifically, we chose fused silica (Spectrosil 2000) of 10 mm × 10 mm × 0.55 mm in size coated with a metal film deposited by electron beam evaporation (Ardenne CS400ES) comprising 10 nm chromium followed on top by 50 nm gold. For the conductive interconnection to the biased sample holder, the substrates are clamped from the top. We note that n‐ and p‐doped monocrystalline silicon substrates were also tested and found to also allow the field‐emission‐based printing process.

As the TEBID system, an AFMinSEM (nano analytik GmbH,^[^ ^30^ ^]^ Figure 1a) was utilized. The AFM was installed into an SEM equipped with a focused ion beam (FIB) column and a GIS (Helios600i DUALBeam system from Thermo Fisher,^[^ ^31^ ^]^), as shown in Figure 1b.

Naphthalene was used as the precursor species generated from a heated solid‐state target. The outgassing carbon components are injected directly into the beam path via a cannula (Figure 1c). This configuration allows very localized gas injection without a major vacuum drop in the chamber but greatly restricts the available installation space. The exit end of the injection cannula is150 µm away from the TEBID writing spot on the sample.

The AFMinSEM system used allows coarse positioning of the cantilever head over 18 mm × 18 mm × 10 mm in *X‐Y‐Z*. In addition, the system provides a bottom scanner with an adjustment range of 60 µm × 60 µm × 20 µm and a theoretical resolution of 0.4 nm × 0.4 nm × 0.2 nm (*X‐Y‐Z*) for typical AFM measurements. The brass sample holder is gold‐plated (10 µm bright nickel and 1 µm gold) and can be connected to an external voltage source (bias voltage of 0 V up to 100 V, DC) with the conductive field‐emission tip grounded. The coarse positioner was used to define the writing spot on the sample in the *X‐Y* direction and was switched off during the 3D writing sequence. The *X‐Y* axis of the bottom scanner defines the beam path in the horizontal direction while the *Z* axis regulates the tip‐sample distance to a constant current setpoint by means of a proportional‐integral feedback loop during pattern generation.

### 2.2 Proof of Concept

**Figure 2:**
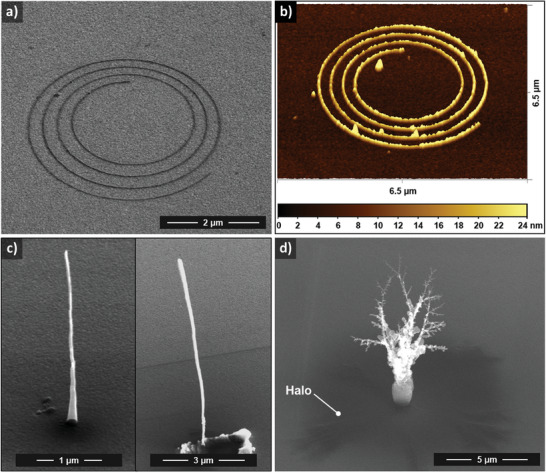
Deposition of 2D and 3D nanostructures from an AFM tip using the TEBID method with a naphthalene precursor a) SEM of a spiral deposited using 60 V bias, 35 pA emission current, IG = 70–120; b) AFM image of the spiral in (a), revealing line width of ≈70 nm (FWHM) and feature height of ≈20 nm; c) SEM of two nanowire‐like structures deposited using the same TEBID parameters as in (a), but created in *Z* direction. The right side shows a structure height of up to 10 µm with a diameter of ≈100 nm, but also shows an example of a tip crash occurring prior to the actual deposition; d) SEM of a tree‐like 3D nanostructure, achieved by increasing the current setpoint to 180 pA with simultaneous reduction in the retract speed toward IG = 3, the described parasitic deposition around the structures is labeled as a halo in the image.

Based on a previously established parameter range for stable emission from the cantilever tip at ambient pressure,^[^ ^29^, ^30^ ^]^ a spiral trajectory was used to create a carbon deposit with a spiral morphology (**Figure** **2**a). The TEBID parameters used were 60 V bias voltage, 35 pA emission current setpoint, writing speed 100 nm s^−1^, and integral gain (IG) manually regulated in the range of IG = 70–120. The chosen geometry demonstrates the planar deposition feasibility of the system, requiring continuous precursor decomposition, patterning trajectories in two directions of motion, and control of the *Z*‐height to a constant setpoint. The deposit line width is determined by the dose, which is primarily controlled by the writing speed. For further evaluation of the spiral deposit, we carried out AFM measurements as shown in Figure 2b. The measured line width was ≈70 nm full width at half maximum (FWHM), with a height of ≈20 nm.

To create a 3D nanostructure, the tip or sample must be moved in a controlled manner in the vertical *Z* direction over the range of several micrometres, due to the continuously changing substrate surface during the deposition. However, this poses a considerable challenge for system control, as the emission current depends strongly on the distance between the sample surface and the tip, requiring position control in the sub‐nanometre range. Furthermore, the electric field distribution must be considered to fully understand the electron interaction with the deposit. We note that the controller and pattern editor of the AFMinSEM system used in the present work is limited to absolute point‐based positioning. Thus the direct import of complex 3D files such as those used in modern CNC machines and 3D printers was not yet possible, for which path‐based trajectories would be required. Detailed modeling of the TEBID process is another important factor to address to enable controlled and reproducible 3D printing using this method. Here it must be considered that the TEBID process is highly dynamic. For example, the field emission process is likely to originate statistically from different emission points, which can also change depending on the nanostructure being deposited and due to possible contaminants on the tip. Modelling should therefore ultimately include the complex and variable interaction between the nanostructure and the tip during the growth. Nevertheless, in a very simplified view, the tip and the sample can be modeled in their initial state as a point‐plate configuration, resulting in an inhomogeneous electric field distribution. The degree of inhomogeneity is linked to the absolute *Z* distance and will directly affect the electron interaction probability and thus the deposition. As soon as the initial nanostructure is nucleated underneath the tip, the tip‐plate configuration steadily transforms into a point‐point‐like interaction, affecting the subsequent growth and morphology accordingly.

From the above‐simplified perspective, it can be expected that nanostructures with a well‐defined nanowire‐like geometry can be realized, especially over smaller *Z*‐distances. Nevertheless, further factors contribute, such as the integral gain (IG) value. A higher IG value results in faster response of the controller to reach the setpoint by retracting the sample at constant bias voltage. However, this can also lead to unwanted oscillations of the tip in the Z direction, and thus to an unstable emission current and inhomogeneous growth. Such instabilities are to be expected, in particular for higher bias voltages and current setpoints, both of which are linked to *Z* movements of the tip within the control system. The absolute emission current setpoint is also a critical parameter in other respects. First, since stable emission is required, the overall current range in our case is limited to 0 to 200 pA, as reported previously.^[^ ^32^ ^]^

Second, the selected emission current correlates with the interaction probability and thus with growth rate.

By assuming that the surface diffusion of ad‐atoms is for nanowire growth just as important as for thin film growth, higher growth rates are expected to potentially yield morphologies that are less well defined.

In accordance with these basic assumptions and using otherwise identical TEBID parameters to those employed for the growth of the spiral structure, a nanowire with a height of up to 10 µm was grown by controlled *Z* motion of the tip over this vertical distance (Figure 2c). However, despite using the same parameter set, reproducibility was not guaranteed, i.e., frequently no deposit was grown. We primarily attribute this to the high integral gain value and the related tip instabilities. Improved reliability was achieved by significantly reducing the integral gain value while simultaneously increasing the emission current setpoint. Yet an increase in the emission current by over a factor of 5 (from 35 pA up to 180 pA) yielded less defined (or more complex) 3D structures with a highly branched corona discharge appearance, as shown in Figure 2d. Note, the integral gain value was already lowered in this case for the sake of stability to IG = 3. The branched growth is likely due to charging of the carbon material during the deposition, causing the aggregation of polarized/ionized precursor molecules along electric field gradients.^[^ ^33^, ^34^ ^]^

These first experiments demonstrate that 2D and 3D carbon deposits can be created by locally decomposing a gaseous naphthalene precursor underneath an electron field‐emission nanoscale scanning tip using the TEBID method.

### 2.3 TEBID Parameter Evaluation

The characteristic system control parameters to be evaluated are the bias voltage and the integral gain IG. Since the reproducible creation of structures by TEBID was only possible for relatively high emission current setpoints, this value was mainly kept constant at 100 pA, unless otherwise noted.

**Figure 3:**
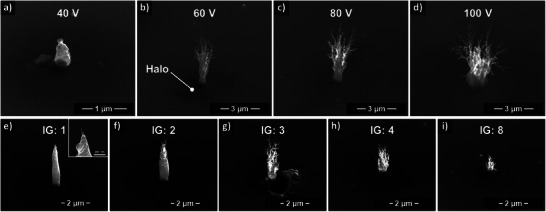
TEBID parameter study of the effect of bias voltage in a–d), and the integral gain IG in e–i), with constant emission current setpoint at 100 pA.

To first explore the impact of the applied bias voltage, the voltage was varied from 40 to 100 V in four steps using an IG value of 3. **Figure** **3**a–d shows SEM images of the corresponding 3D structures, which are representative for this particular parameter set. As the bias voltage is a key parameter determining the electron emission current, the fixed current setpoint and the linked current‐based control results in an increased tip‐sample distance. As shown here, and in alignment with the basic assumptions stated previously, the 3D nanostructure growth clearly depends on the tip bias voltage for a fixed current setpoint. At 60 V, nanostructure branching occurs, becoming more prominent upon further increasing the bias. The width of the base of the structure also increases over the 60–100 V bias range tested. These trends could be due to the increase in tip‐sample distance for fixed current setpoint, which in turn affects the electric field distribution and thereby the deposit morphology, with a tendency toward more irregular growth.

Varying the integral gain value was investigated in the range of IG = 1 to 8 (Figure 3e–i). The effect of the IG value is highly specific to the system, but essentially it represents the travel speed with which the system attempts to readjust the tip‐sample distance in the *Z*‐direction when the current setpoint deviation becomes too large. The IG values correspond approximately to the maximum travel speeds as follows: IG = 1–175 nm s^−1^, IG = 2–345 nm s^−1^, IG = 3–505 nm s^−1^, and IG = 4–680 nm s^−1^. Since it was not possible to perform an exact interferometric measurement to determine the system specific values of the driving speed, the set IG value is instead used as a sufficiently characteristic value. The experimental results show that the degree of branching increases with increasing IG value, which can be a direct result of the increased travel speeds. Furthermore, the total volume of the structures and elongation in the *Z*‐direction appear to be suppressed for larger IG values, such as 4 and 8. This might be linked to severe tip oscillations and associated emission current instabilities.

Besides the tree‐like nanostructure growth with filigree branches, we also observed halo deposits around the base of some of the structures (cf. Figure 3b, for example). This could be parasitic carbon deposition caused by forward‐scattered electrons from the deposit and backscattered electrons from the surrounding substrate.^[^ ^35^ ^]^ In a subsequent study,^[^ ^36^ ^]^ Plank et al. describe for EBID a linear increase in the width of the parasitic halos with increasing height of the deposit, attributed to the predominant influence of the forward scattered electrons. These electrons, which can escape the deposit structure in the direction of the sample surface, still have sufficient energy to cause decomposition of the precursor molecules adsorbed on the surface. However, the influence of charging or thermal effects cannot be excluded at the present time.

### 2.4 Tip Effects and Additional Deposit Analysis

**Figure 4:**
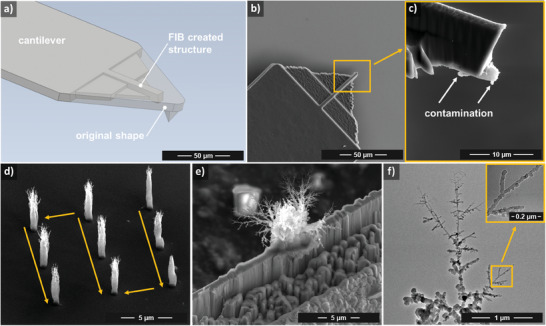
a) Schematic showing tip‐less microcantilever geometry formed by FIB milling; b) SEM view of the final tip‐less cantilever; c) side view of the end of the microcantilever used for structuring, showing parasitic deposits; d) array of nanostructures formed by TEBID using the 50 nm UNCD‐coated tip‐less microcantilever with optimized writing path; e) TEBID structure fabricated onto a 1 µm thick FIB‐milled wedge; f) bright‐field TEM analysis of the deposit with magnified insight into the marked area.

The scanning tip itself also influences the TEBID growth, with the material and morphology of the tip affecting the electron emission. Another critical point is potential mechanical contact of the tip with the sample surface, which can change the shape of the tip, lead to its contamination, and damage the substrate/deposit structure (cf. **Figures** 3g and **4**c). Changes to the tip also directly affect the electric field distribution and hence the electron‐precursor interaction, and in turn, the nature of the localized deposition. At present, such variations limit the reproducibility and optimization of the TEBID approach and will be the subject of future studies.

Although one might think that ultra‐sharp tips are the most suitable tip configuration, it has recently been shown that blunter tips (tip radius ≥ 50 nm) also show field‐emission that can be used for high‐resolution field‐emission scanning probe lithography.^[^ ^37^, ^38^ ^]^ In the cited work, scanning tips coated with ultra‐nano crystalline diamond (UNCD) were used. Compared to conventional silicon‐based tips, such coatings are expected to improve tip durability as well as the field‐emission properties due to grain boundary effects.^[^ ^39^, ^40^, ^41^, ^42^ ^]^ Therefore, in the present work, tip‐less microcantilevers with UNCD coating were also tested.

The tip‐less shape investigated here was obtained by FIB milling to remove the tip from an original microcantilever and form a simple cantilever beam (Figure 4a–c). While sharp tips are expected to emit electrons from a highly localized emission site at the tip apex, tip‐less microcantilevers with UNCD coating would be expected to emit stochastically from several emission spots, lowering the TEBID resolution. Furthermore, the tip‐less geometry would result in a plate‐point electric field distribution, influencing the deposit morphology. However, as we show here, neither resolution nor deposit morphology appear to be significantly affected by the tip‐less shape.

With respect to process stability and reproducibility, a flat (tip‐less cantilever) or blunt tip in fact appears to be beneficial compared to sharp tips. An example of a set of carbon nanostructures obtained using the tip‐less 50 nm UNCD‐coated cantilever is shown in Figure 4d, obtained using a bias voltage of 60 V, an emission current setpoint of 180 pA, and an integral gain value of 2. The *X‐Y* trajectory employed is indicated in the figure with the arrows and was defined in this manner to minimize both the overall time required (compared to a line‐by‐line trajectory) and to prevent interaction of the probe with the previously created structures. We note that the contamination on the cantilever marked in Figure 4c is due to parasitic deposits formed during the TEBID process.

To gain further insight into the mechanism of the TEBID printing process, comprehensive understanding of not only the deposit morphologies but also the deposit compositions and structure is required. This information is crucial for applications targeting deposits with specific functionalities. With dimensions down to the nanometer scale (such as the filigree branched structures grown here), high‐resolution materials analysis techniques are needed. To this end, we have used transmission electron microscopy (TEM) for initial investigations in this area. To allow inspection by the TEM method, TEBID structures were deposited onto a TEM‐compatible custom copper wedge substrate prepared by FIB milling (Figure 4e). The fact that TEBID could be successfully performed onto the wedge further demonstrates the control of the deposit location, as well as the ability to deposit onto non‐planar substrates. A bright‐field TEM view of the end portion of one of the branches of the tree‐like carbon nanostructure is shown in Figure 4f. Imaging was performed using a TitanX TEM operated at 300 kV. The inset shows a higher magnification view of a single branch showing a branch width of ≈20 nm. The filigree branches appear to be amorphous in nature, although further analysis is required to investigate the possibility of local crystalline regions. In any event, Kulkarni et al. have shown that amorphous carbon deposits can be transformed into structures with crystallographic phases by means of sample heating up to 450 °C.^[^ ^43^ ^]^ Such a method would be interesting to pursue to post‐tune the material properties of TEBID structures.

## 3 Summary

In this study, field‐emission of low‐energy (<100 eV) electrons from scanning probe tips was used to trigger the highly localized decomposition of a gaseous naphthalene precursor. We successfully fabricated a variety of 2D and 3D nanostructures by adjusting system parameters such as bias voltage, emission current setpoints, and integral gain, and discussed their impact on the TEBID process. The fabrication of high aspect ratio nanowire‐like structures and branched tree‐like nanostructures was demonstrated. In addition to ultra‐sharp silicon tips, tip‐less microcantilevers coated with ultra‐nanocrystalline diamond were also investigated. The latter were found to increase the reliability and hence the reproducibility of the TEBID process. Using such “blunt” tips, we fabricated arrays of nanostructures on planar substrates and deposits at specific locations on non‐planar wedge substrates. The presented results deliver proof of concept of tip‐induced 3D printing on the nanoscale using field‐emission scanning probes, providing inspiration for further research and development in this area. Future research and development toward a standalone TEBID 3D printing platform should investigate a wider range of precursor chemistries, analyze the deposit compositions and structures in greater detail, optimize writing fields and throughput, enable complex path‐based 3D trajectories, and overall improve and extend system control.

## Conflict of Interest

The authors declare no conflict of interest.

## Supporting information



Supporting Information

## Data Availability

The data that support the findings of this study are available from the corresponding author upon reasonable request.
