# Supplementary material for: Tip‐Induced 3D Printing on the Nanoscale with Field Emission Scanning Probes
Source: Small. 2024 Dec 18;21(5):2409035. doi: 10.1002/smll.202409035 (PMC11798345; doi:10.1002/smll.202409035)
Supplement: Supplementary file 1 — Supporting Information [file SMLL-21-2409035-s001.pdf]

NANO · MICRO
small

Supporting Information

for *Small*, DOI 10.1002/smll.202409035

Tip-Induced 3D Printing on the Nanoscale with Field Emission Scanning Probes

*Mathias Holz, Martin Hofmann, Frances I. Allen, Christoph Weigel and Steffen Strehle**

Supplementary information:

Tip-induced 3D printing on the nanoscale with field emission scanning probes

Mathias Holz[1], Martin Hofmann[1], Frances I. Allen[2,3], Christoph Weigel[1], and Steffen Strehle[1]*

[1] Technische Universität Ilmenau, Institute of Micro- and Nanotechnologies MacroNano®, Microsystems Technology Group, Max-Planck-Ring 12, 98693 Ilmenau, Germany
[2] Department of Materials Science and Engineering, University of California, Berkeley, California 94720, USA
[3] National Center for Electron Microscopy, Molecular Foundry, Lawrence Berkeley National Laboratory, Berkeley, California 94720, USA

*E-mail: steffen.strehle@tu-ilmenau.de

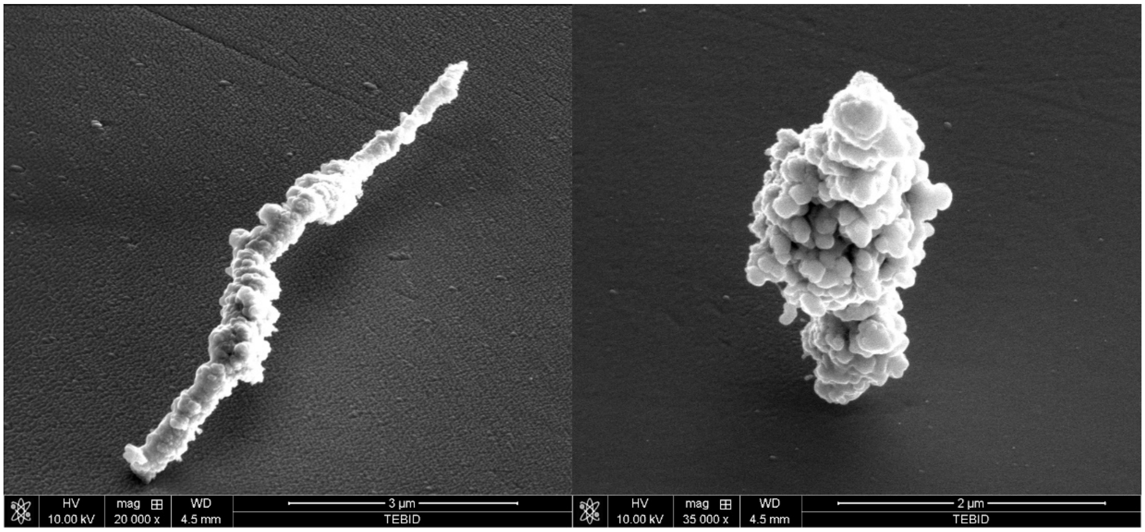

Scanning electron microscopy (SEM) images of 3D printed, organometallic pillar-type microstructures using trimethyl(methylcyclopentadienyl)platinum(IV) as the precursor for the tip-based electron beam induced deposition (TEBID) process; on the left, the TEBID parameters used were 30 V bias voltage, 80 pA current setpoint, IG = 2 integral gain; on the right, 20 V bias voltage, otherwise identical parameters.
